# Coordination of consolidated bioprocessing technology and carbon dioxide fixation to produce malic acid directly from plant biomass in *Myceliophthora thermophila*

**DOI:** 10.1186/s13068-021-02042-5

**Published:** 2021-09-23

**Authors:** Jingen Li, Bingchen Chen, Shuying Gu, Zhen Zhao, Qian Liu, Tao Sun, Yongli Zhang, Taju Wu, Defei Liu, Wenliang Sun, Chaoguang Tian

**Affiliations:** 1 Key Laboratory of Systems Microbial Biotechnology, Tianjin Institute of Industrial Biotechnology, Chinese Academy of Sciences, Tianjin, 300308 China; 2 National Technology Innovation Center of Synthetic Biology, Tianjin, 300308 China; 3 University of Chinese Academy of Sciences, Beijing, 100049 China

**Keywords:** *Myceliophthora*, Metabolic engineering, CBB cycle, CO_2_-fixation, Plant biomass, Malic acid

## Abstract

**Background:**

Consolidated bioprocessing (CBP) technique is a promising strategy for biorefinery construction, producing bulk chemicals directly from plant biomass without extra hydrolysis steps. Fixing and channeling CO_2_ into carbon metabolism for increased carbon efficiency in producing value-added compounds is another strategy for cost-effective bio-manufacturing. It has not been reported whether these two strategies can be combined in one microbial platform.

**Results:**

In this study, using the cellulolytic thermophilic fungus *Myceliophthora thermophila*, we designed and constructed a novel biorefinery system DMCC (Direct microbial conversion of biomass with CO_2_ fixation) through incorporating two CO_2_ fixation modules, PYC module and Calvin–Benson–Bassham (CBB) pathway. Harboring the both modules, the average rate of fixing and channeling ^13^CO_2_ into malic acid in strain CP51 achieved 44.4, 90.7, and 80.7 mg/L/h, on xylose, glucose, and cellulose, respectively. The corresponding titers of malic acid were up to 42.1, 70.4, and 70.1 g/L, respectively, representing the increases of 40%, 10%, and 7%, respectively, compared to the parental strain possessing only PYC module. The DMCC system was further improved by enhancing the pentose uptake ability. Using raw plant biomass as the feedstock, yield of malic acid produced by the DMCC system was up to 0.53 g/g, with ^13^C content of 0.44 mol/mol malic acid, suggesting DMCC system can produce 1 t of malic acid from 1.89 t of biomass and fix 0.14 t CO_2_ accordingly.

**Conclusions:**

This study designed and constructed a novel biorefinery system named DMCC, which can convert raw plant biomass and CO_2_ into organic acid efficiently, presenting a promising strategy for cost-effective production of value-added compounds in biorefinery. The DMCC system is one of great options for realization of carbon neutral economy.

**Supplementary Information:**

The online version contains supplementary material available at 10.1186/s13068-021-02042-5.

## Background

Concern over global climate charge and unstable petroleum supply has led to a greater emphasis on renewable energy sources to decrease reliance on fossil fuels [1, 2]. As a non-edible plant material, lignocellulosic biomass has long been recognized as a potential sustainable source for many industrial applications and green biosynthesis of biofuels and commodity chemicals. However, because of the recalcitrant nature of lignocellulose to enzymatic hydrolysis, the costs of conversion of insoluble lignocellulosic materials to fermentable sugars represent a significant barrier to the production of cost-competitive biofuels [3, 4]. Consolidated bioprocessing (CBP), featuring the hydrolysis and fermentation in a single process step without adding any extra cellulases, is widely recognized as a promising strategy for cost-effective production of plant biomass-derived biofuels and chemicals [5]. CBP entails microbial engineering by functional expression of cellulases in a fermentative organism, or incorporation of the desired product-synthesis pathway into a cellulase-producing organism [3, 6]. Recently, cellulolytic organisms, such as *Trichoderma* [7], *Aspergillus*, *Clostridium* [8]*,* and *Myceliophthora* [9, 10] have been tried as CBP strains for direct conversion of plant cell wall materials into biofuels and biochemicals, including ethanol [11], isobutanol [12], itaconic acid [13], and malic acid [9, 10].

CO_2_ is also a potentially scalable raw material to produce sustainable fuels and chemicals that could be alternatives to petroleum products. Fixing and channeling CO_2_ into the central carbon metabolism of industrial microbes has potential to reduce the CO_2_ level in the environment and increase carbon efficiency in the production of value-add compounds, for example, production of succinic or acetic acid from methanol and CO_2_ [14]. Several CO_2_ fixation pathways, including the Calvin–Benson–Bassham (CBB) cycle, the 3-hydroxypropionate bicycle, and methanol condensation pathways, have been introduced into heterotrophic organisms, such as *Escherichia coli*, *Saccharomyces cerevisiae*, *Pichia pastoris*, and *Methylobacterium extorquens* [15–22].

The CBB cycle, also known as the reductive pentose phosphate cycle, is the most dominant CO_2_ fixation pathway of the seven known natural alternatives [23–25]. The CBB cycle employs 11 enzymes to complete autotrophic CO_2_ fixation. Most of these enzymes are also involved in central metabolism, including glycolysis and the pentose phosphate pathway [26]. Ribulose-1,5-bisphosphate carboxylase–oxygenase (RuBisCO) and phosphoribulokinase (PRK) are the two key enzymes of the CBB cycle. PRK catalyzes phosphorylation of ribulose-5-phosphate, a normal intermediate of pentose phosphate pathway, to ribulose-1,5-biphosphate. RuBisCO is the one enzyme that is specific to the CBB cycle, and catalyzes the carboxylation of ribulose-1,5-bisphosphate with CO_2_ to generate two molecules of 3-phosphoglycerate [27]. Functional overexpression of genes encoding RuBisCO and PRK in *E. coli* resulted in significantly decreased release of CO_2_ [20, 22, 28] and the biosynthesis of sugar from CO_2_ [29]. Based on construction of RuBisCO-dependent *E. coli*, a RuBisCO mutant with higher activity and better solubility was selected [30, 31]. Recycling CO_2_ into the central metabolic network is a promising approach for expanding the use of CO_2_ fixation to improve the yield of target metabolites. In *S. cerevisiae*, parts of the CBB cycle have been integrated into the metabolic network to enable the use of CO_2_ as an additional electron acceptor for the reoxidation of NADH and simultaneous recycling of CO_2_ released from the decarboxylation of pyruvate, resulting in increased productivity and yield of ethanol [17, 21, 32]. Recently, a non-native CBB cycle has been introduced into heterotrophic organisms to generate autotrophic or mixotrophic organisms, coupling of modification of central metabolic pathway. Antonosky et al. engineered *E. coli* to hemi-autotrophically grow on CO_2_, with reducing power and energy from oxidation of pyruvate [29]. In follow-up studies, *E. coli* and *P. pastoris* were converted into autotrophs, which could incorporate CO_2_ into biomass, respectively, using formate or methanol as the energy source for a heterologous CBB cycle [18, 33]. Pentoses, including xylose and arabinose, are metabolized by pentose phosphate pathway, where the intermediate ribulose-5-phosphate can serve as the substrate of PRK. Previously, xylose and arabinose were used as the feedstock to drive CO_2_-fixation by the CBB cycle in *E. coli* and *S. cerevisiae* [17, 20, 32].

Malic acid is widely used in the food industry as the acidulant and flavor enhancer and it was selected as one of the 12 most important building block chemicals available from renewable biomass by the US Department of Energy in 2004. Studies evaluating microbial malate production have attracted much industrial attention. Four native metabolic pathways that produce malic acid from glucose have been identified and analyzed [34]. The reductive tricarboxylic acid (rTCA) pathway, with theoretical yield of 2 mol malic acid/mol glucose, is considered the most efficient pathway, because of the CO_2_ fixation during the carboxylation reaction of pyruvate to oxaloacetate catalyzed by pyruvate carboxylase [34]. Filamentous fungi offer great potential advantages in the use of complex carbon sources and production of organic acids at high concentration with yield near the theoretical maximum, and several such species have been engineered as cell factories for producing malic acid, including *Aspergillus*, *Penicillium*, *Rhizopus*, and *Myceliophthora* [35–38].

The thermophilic filamentous fungus *Myceliophthora thermophila* (synonym *Thermothelomyces thermophilus*), which is able to secrete a large amount of hydrolytic enzymes and grow robustly on cellulosic materials, is exceptionally attractive for biorefinery construction [39, 40]. A suite of molecular biology tools, including CRISPR/Cas9 method, are available for *M. thermophila*, which allow rational genetic engineering. Previously, we enhanced the synthetic pathway and export system of malic acid in *M. thermophila* and the resultant transformant was able to produce malic acid by direct conversion of hemicellulose or cellulose without adding extra hydrolase [9]. The major components of plant cells are made from hexose (glucose) and pentoses (xylose and arabinose). Pentose is catabolized via the pentose phosphate pathway and can actuate CO_2_-fixation during the CBB cycle.

**Fig. 1:**
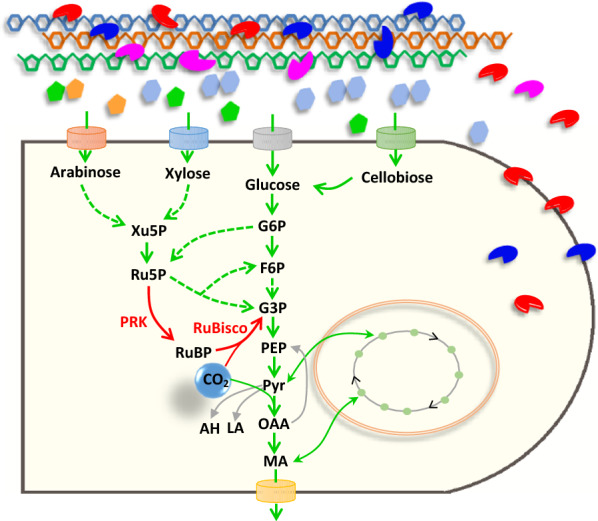
Overall scheme of synergistic direct conversion of lignocellulosic biomass with CO_2_-fixation to produce organic acid in *Myceliophthora thermophila*. *G6P* glucose-6-phosphate, *F6P* fructose-6-phosphate, *G3P* glycerate-3-phosphate, *PEP* phosphoenolpyruvate, *Pyr* pyruvate, *OAA* oxaloacetic acid, *MA* malic acid, *Xu5P* xylulose-5-phosphate, *Ru5P* ribulose-5-phosphate, *AH* acetaldehyde, *LA* lactic acid

In this study, the CBB cycle enzymes RuBisCO and PRK were introduced into the cellulolytic fungus *M. thermophila* to form a novel biorefinery system—called DMCC (Direct microbial conversion of biomass with CO_2_ fixation) here—to produce a bulk chemical (malic acid) from plant biomass and CO_2_ (Fig. 1). The function of the heterologous CBB cycle was demonstrated by growth phenotype, ^13^C-tracer analysis, and increased production of malic acid. Moreover, branches from the malate synthesis pathway were deleted by gene knock-in technology mediated through the CRISPR/Cas9 system, and genetic manipulation for improved uptake of pentose was performed. The combined effects of these manipulations led to improved malic acid titer and yield directly from plant biomass (corncob) and CO_2_, exhibiting the latest progress in CBP strategy.

## Results

### Heterologous expression of genes encoding CBB cycle enzymes in *M. thermophila*

Previously, we performed metabolic modification in *M. thermophila* to generate strain JG207, which can produce malic acid using plant lignocellulose as the carbon source [9]. Efficient utilization of the main components of lignocellulose (glucose, xylose, and arabinose) is a prerequisite for improvement in three major indices of performance (titer, yield and productivity) in the biological production of plant cell-derived biochemicals. In microbial cells, xylose and arabinose are metabolized by pentose phosphate pathway, which can provide the substrate (ribulose-5-phosphate) for PRK of the CBB cycle. The genes encoding CBB cycle enzymes—RuBisCO and PRK—have been introduced into heterotrophic microbes to recycle CO_2_ and driven by pentose to improve production of target metabolites [17, 29, 32]. It was reported that prokaryotic form-II RuBisCOs are encoded by a single structural gene [27]. The RuBisCO gene from *R. rubrum* (*cbbM*) has been functionally overexpressed in *M. extorquens*, *E. coli* and *S. cerevisiae* [17, 19]. Therefore, to improve malate production by *M. thermophila*, *cbbM* was integrated into the genome of strain JG207, together with *prk* from *S. oleracea*, under the control of the strong constitutive promoters of *gpdA* and *pdc*, respectively. After confirmation of the presence of the transgenes by PCR analysis, physiological characterization of the resultant strain, named as CP-1, was conducted using xylose or arabinose as the carbon source.

**Fig. 2:**
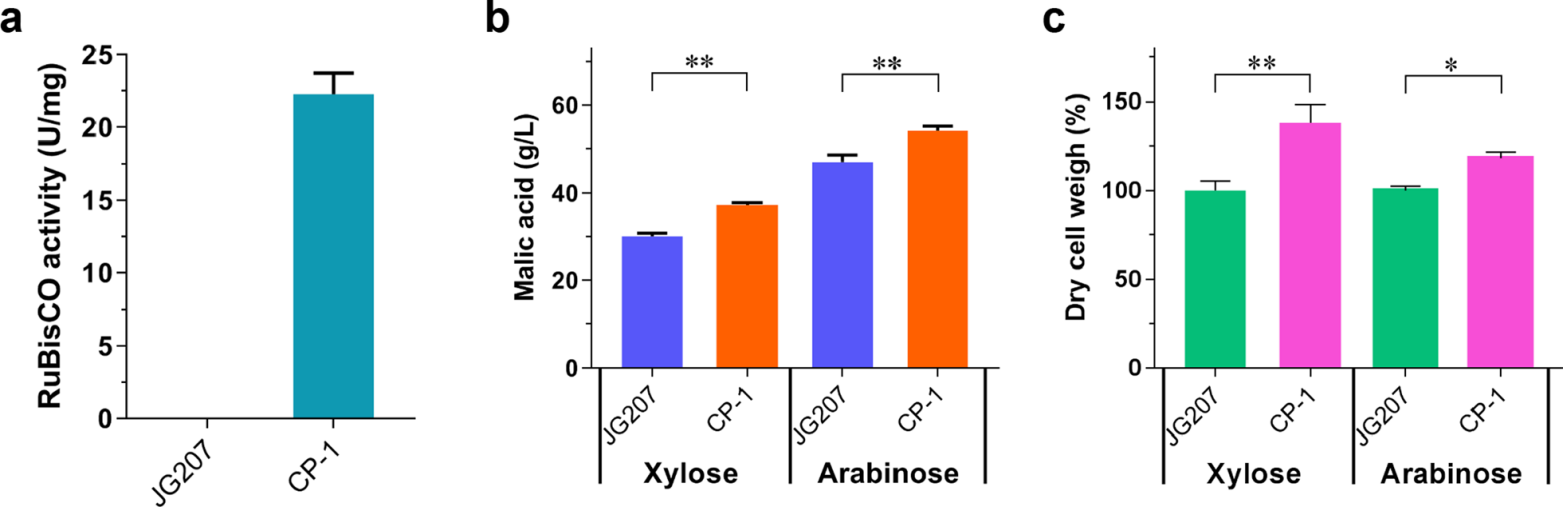
Physiological characterization of *M. thermophila* strain CP-1 overexpressing genes encoding ribulose-1,5-bisphosphate carboxylase/oxygenase (RuBisCO) and phosphoribulokinase (PRK). **a** RuBisCO activity in *M. thermophila* CP-1. **b** Titers of malic acid in culture of strain CP-1 when grown on xylose or arabinose after 8 days in shaking flasks. **c** Dry cell weight of culture of strain CP-1 grown on xylose or arabinose for 4 days. The values and error bars represent means and standard deviations

RT-qPCR analysis indicated that six copies of *prk* and eight copies of *cbbM* were integrated into the genome of strain CP-1 (Additional file 2: Fig. S2). RuBisCO was active in strain CP-1 when *cbbM* was overexpressed. The RuBisCO activity in the crude extract of this strain achieved 22.3 U/mg protein, while in the parent strain JG207, the activity was undetectable (Fig. 2a). As shown in Fig. 2, simultaneous overexpression of RuBisCO and PRK contributed to malate production from xylose or arabinose. Starting with 75 g/L carbon source, the titer of malic acid reached 37.3 g/L and 54.1 g/L from xylose and arabinose, respectively, 24% and 15% increases compared with parental strain JG207 (malate titers 30 g/L and 47 g/L from xylose and arabinose, respectively). Moreover, increases in cell dry weight of strain CP-1 on xylose (1.38-fold) and arabinose (1.18-fold) were confirmed, compared with strain JG207. These results indicated that integration of the CBB cycle enzymes into the carbon metabolic pathway of a filamentous fungus was beneficial for malate production and cell growth when using pentoses as the feedstock.

### Simultaneous CO_2_-fixation and branch pathway disruption were propitious to the production of malic acid

Based on the observations described above, we found that the engineered strain containing CBB cycle enzymes had an advantage in malate production. We then applied another metabolic engineering strategy: disruption of the branch points of the synthetic pathway of the target chemical (malic acid) while overexpressing the CBB cycle enzymes. Previous study indicated that in microbes, pyruvate decarboxylase and lactate dehydrogenase, encoded by *pdc* and *ldh*, respectively, use pyruvate as a substrate. These reactions limit organic acid production via the rTCA pathway [9, 41]. Moreover, phosphoenolpyruvate (PEP) carboxykinase (PCK) functions on inverse reaction of PEP to oxaloacetate, the precursor for malate synthesis, in *M. thermophila*, and has been considered as a target of metabolic engineering to improve production of malic acid [9]. In *M. thermophila*, the genes *pdc*, *ldh*, and *pck* show high expression levels in response to various carbon sources [40, 42]. Therefore, here, *prk*, *cbbM*, and the selective marker gene *neo* were integrated into the *ldh*, *pdc*, and *pck* loci, respectively, in the genome of strain JG207, using the CRISPR/Cas9 system. Thus, the CBB cycle enzymes would be heterologously expressed and branch pathways were simultaneously deleted, forming strain CP-51.

**Fig. 3:**
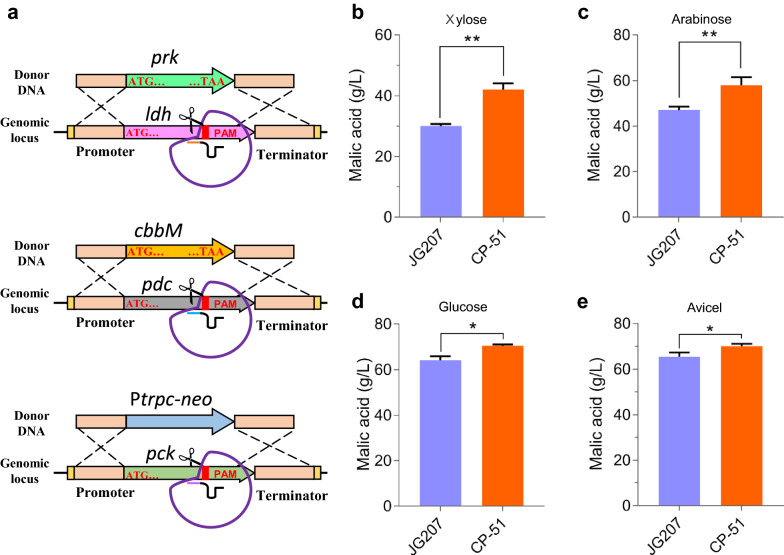
Combined effects of Calvin–Benson–Bassham (CBB) cycle and disruption of branch pathway genes on production of malic acid. **a** Schematic representation of integration of *prk*, *cbbM*, and selective marker gene *neo* into the *ldh*, *pdc*, and *pck* loci, respectively, of the *M. thermophila* genome using the CRISPR/Cas9 system. Titers of malic acid produced by strain CP-51 grown on (**b**) xylose, (**c**) arabinose, (**d**) glucose, and (**d**) Avicel. Titer of malic acid was determined after 8 days of fermentation. The values and error bars represent means and standard deviations

When grown on xylose, strain CP-51 displayed 1.40-fold and 1.13-fold increases in malate production compared with the values for strains JG207 and CP-1, respectively. The titer of malic acid was up to 42.1 g/L (Fig. 3b). The dry cell weight of strain CP-51 showed a 59% increase compared with that of the parental strain JG207 (Additional file 2: Fig. S3). Consistently, strain CP-51 was superior to strains JG207 and CP-1 in production of malic acid using arabinose, glucose, and even cellulose (Avicel) as the carbon source. Titers of malic acid produced by strain CP-51 grown on arabinose, glucose and Avicel in flask culture were improved to 58.0 g/L, 70.4 g/L, and 70.1 g/L, respectively. The corresponding yields were 0.77 g/g, 0.94 g/g and 0.93 g/g carbon source, respectively. In addition, due to the disruption of *pck* and *ldh*, ethanol and lactate were undetectable in the culture. These results revealed that the combination of integration of CBB cycle enzymes and disruption of branch pathways contributed to further improvement of malate production from lignocellulose-derived sugars.

### Confirmation of CO_2_-fixation by ^13^C-tracer analysis

**Fig. 4:**
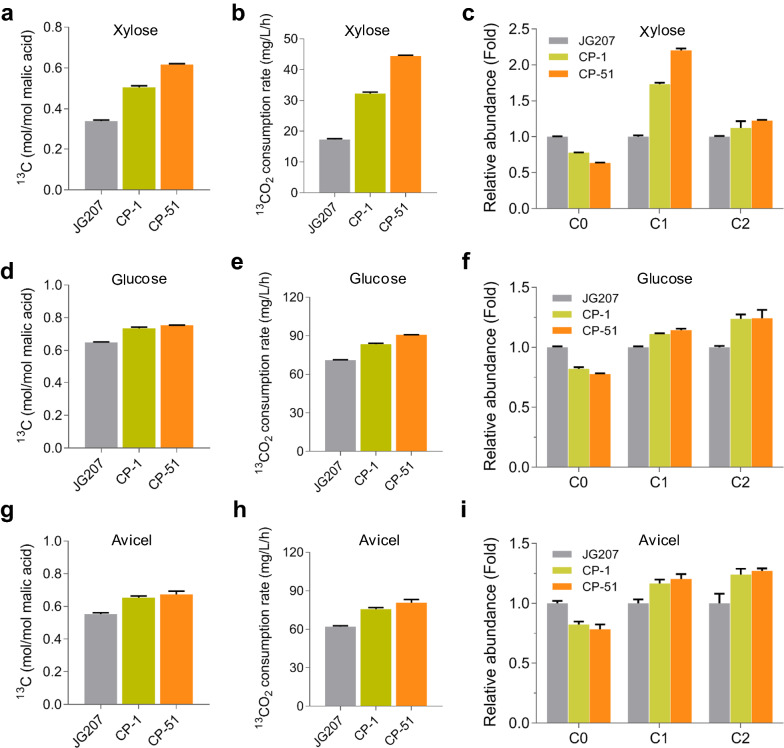
^13^C-tracer analysis of CO_2_-fixation. **a**, **d**, and **g**^13^C content in malic acid produced by strains JG207, CP-1, and CP-51, when grown on various carbon sources for 8 d. **b**, **e**, and **h** The average rates of fixing and channeling ^13^CO_2_ into malic acid. **c**, **f**, and **i** Relative abundance of mass isotopomers of malic acid in culture of strains CP-1 and CP-51, with that in strain JG207 as the control. C0 represents malic acid without a ^13^C atom; C1 represents malic acid with one ^13^C atom; C2 represents malic acid with two ^13^C atoms. **a**–**c** Xylose, **d**–**f** glucose, **g**–**i** Avicel. Grey, strain 207; olivine, strain CP-1; orange, CP-51. The values and error bars represent means and standard deviations

To confirm CO_2_ fixation during production of malic acid, ^13^C-tracer analysis was performed to detect the relative abundances of malic acid with ^13^C atom after fermentation was over. In malic acid-producing strain JG207, malic acid can be synthesized via rTCA pathway, where pyruvate is catalyzed to oxaloacetate by pyruvate carboxylase (PYC), companied by CO_2_ fixation. In order to estimate the efficiency of CO_2_ fixation by PYC module, ^13^C-tracer analysis was carried out in strain JG207 when grown on xylose, glucose or Avicel. As shown in Fig. 4, the contents of ^13^C molecular were up to 0.34, 0.65, and 0.55 mol/mol malic acid on xylose, glucose, and Avicel, respectively, after 8 days of fermentation. The corresponding average rates of fixating and channeling ^13^CO_2_ into malic acid achieved 17.3, 71.1, and 62.0 mg/L/h. CO_2_ fixation by rTCA. These data indicated that when grown on xylose, efficiency of CO_2_ fixation was lower than that on glucose and Avicel. Malic acid was mainly produced by rTCA pathway under glucose and Avicel conditions.

When using engineered strains CP-1 and CP-51 to produce malic acid, we found that the average rates of fixing and channeling ^13^CO_2_ into malic acid achieved 32.2 mg/L/h and 44.4 mg/L/h on xylose, representing 1.86- and 2.57-fold higher than that of strain JG207 (Fig. 4a). This enhancement led to 50% and 83% increases in contents of ^13^C in malic acid, achieving 0.51 and 0.62 mol/mol malic acid in strain CP-1 and CP-51, respectively (Fig. 4b). The relative abundance of malic acid with one ^13^C atom on xylose was improved by 73.2% and 120%, respectively, compared to the parental strain JG207. Meanwhile, the relative abundance of malic acid with two ^13^C atoms produced by strains CP-1 and CP-51 exhibited 12.3% and 22.7% more than that by strain JG207 (Fig. 4c).

When grown on glucose and cellulose (Avicel), enhancement of CO_2_ fixation was also observed after integration of CBB cycle enzymes (Fig. 4d–g). The average rates of fixing and channeling ^13^CO_2_ into malic acid in strains CP-1 and CP-51 were increased by 17.2% and 27.6%, respectively, and up to 83.3 mg/L/h and 90.7 mg/L/h, respectively, compared to strain JG207 on glucose (Fig. 4e). The corresponding contents of ^13^C in malic acid achieved 0.73 and 0.75 mol/mol malic acid, respectively. When using cellulose (Avicel) as the feedstock, content of ^13^C in malic acid produced by strain CP-51 was up to 0.67 mol/mol malic acid (Fig. 4g), indicating that the production of 1 mol of malic acid is accompanied by the fixation of at least 0.67 mol CO_2._ The average rate of fixing ^13^CO_2_ for synthesizing malic acid achieved 80.7 mg/L/h. In addition, it was observed that ^13^CO_2_ fixation rates of all three strain on glucose and Avicel were much higher than that on xylose. These data indicated that enhancement of CO_2_ fixation resulted from combined effort of pyruvate carboxylation by pyruvate carboxylase and the CBB cycle during malate production. It was noteworthy that the relative abundance of malic acid without ^13^C atoms was high in the cultures of the three strains, which might result from that more malic acid were synthesized by other pathways without CO_2_ fixation on xylose, such as the mitochondrial TCA cycle and the glyoxylate pathway.

### Enhanced pentose uptake facilitates pentose fermentation

**Fig. 5:**
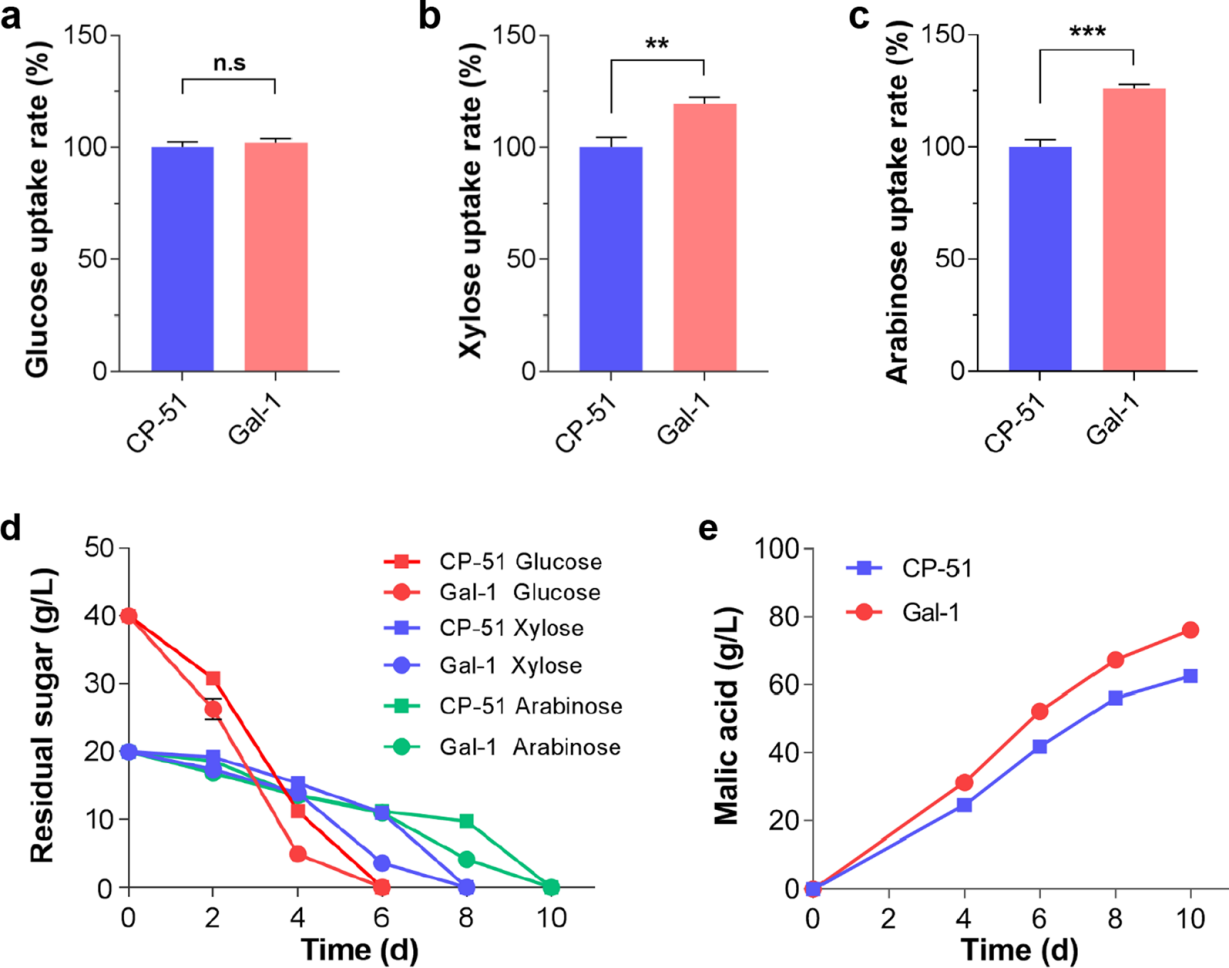
Physiological characterizations of strain Gal-1 overexpressing transporter gene *gla2M*. Substrate transport rates of the mycelia from strain Gal-1 for **a** glucose, **b** xylose, and **c** arabinose. Time-course of sugar consumption (**d**) and malate production (**e**) by strain Gal-1 grown on malate-producing medium supplemented with a mixture of sugars (40 g/L glucose, 20 g/L xylose, and 20 g/L arabinose). The values and error bars represent means and standard deviations

The capability for CO_2_-fixation in engineered strain CP-51 is related to pentose phosphate pathway activity, which can be driven by pentose metabolism. However, xylose use is inhibited by the presence of glucose in the feedstock [43]. Enhancement of pentose uptake can alleviate this inhibition and facilitate improved pentose use and elevated co-fermentation rates of hexose and pentose [44]. Previous study showed that N376F mutation in the Gal2 protein, a galactose/glucose transporter from *S. cerevisiae*, led to improved affinity for xylose and Gal2-N376F becomes a glucose inhibition-free xylose transporter [45]. To further improve the use of pentose to provide the precursor for the CBB cycle, the gene encoding Gal2-N376F (Gal2M), driven by the strong constitutive promoter of *eif* (encoding elongation initial factor), was introduced into strain CP-51. RT-qPCR analysis indicated that eight copies of *gal2M* were integrated into the genome of the resultant strain Gal-1. As we expected, increases in the transport rate of xylose (1.20-fold) and arabinose (1.26-fold) by strain Gal-1 were observed, compared with strain CP-51. The glucose uptake rate was similar to that in the parental strain CP-51 (Fig. 5a–c).

To test the benefits of *gal2M* overexpression on the use of lignocellulosic sugar, strain Gal-1 was incubated in Vogel’s minimal medium supplemented with single or multiple sugars, including glucose, xylose, and arabinose. Enhanced consumption rates of xylose and arabinose were observed in strain Gal-1 compared with strain CP-51 when they were grown with a single sugar as the carbon source. The utilization rate of glucose by strain Gal-1 was similar to that by strain CP-51. When growth in Vogel’s minimal medium supplemented with the mixture of sugars, the level of sugar consumption in strain Gal-1 was considerably higher than that in strain CP-51 (Additional file 2: Fig. S5). When growth on malate-producing medium containing 40 g/L glucose, 20 g/L xylose, and 20 g/L arabinose, which is similar to the components of the hydrolysate of plant biomass, the substrate consumption rate of strain Gal-1 was faster than that of the parental strain CP-51 (Fig. 5d). After 10 days of fermentation, strain Gal-1 achieved a malate titer of 76.1 g/L, a 1.22-fold increase compared with strain CP-51 (62.5 g/L). The corresponding yield was up to 1.01 g/g carbon source in flask cultivation (Fig. 5e). These results indicate that improvement of pentose uptake can facilitate pentose utilization and improve co-fermentation rates of hexoses and pentoses for production of malic acid.

### Conversion of plant biomass and CO_2_ into malic acid using *M. thermophila*

**Fig. 6:**
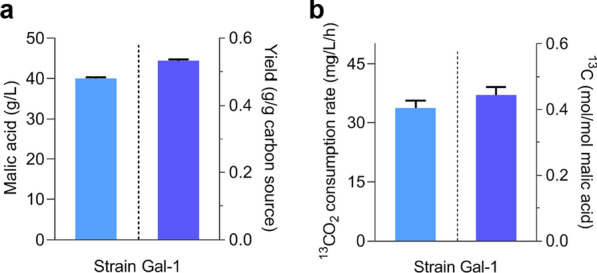
Production of malic acid by *M. thermophila* strain Gal-1 grown on raw corncob. **a** The titer and yield of malic acid was determined after 8 days of fermentation, using raw corncob as the sole carbon source at a final concentration of 75 g/L. **b** The average rate of fixing and channeling ^13^CO_2_ into malic acid and the ^13^C content in malic acid. The values and error bars represent means and standard deviations

In real-word applications, direct utilization of raw plant biomass as the feedstock is the greatest advantage of CBP technique and critical to overcome the remaining barriers to cost-effective production of biofuels and commodities. To test the combined effects of incorporation of the CBB cycle, disruption of branch points of the malate synthesis pathway, and enhancement of substrate uptake on production of malic acid from lignocellulosic biomass, we tested the use of pulverized raw corncob without pretreatment by alkali, acid, or hydrolytic enzymes as the feedstock for malate production. Starting with 75 g/L raw corncob, the titer of malic acid in strain Gal-1 was up to 40 g/L, 10.4% more than that in the original strain JG207 [9]. The yield of malic acid from raw corncob were up to 0.53 g/g total plant biomass (Fig. 6a). These data represent the highest yield of malate production yet reported from raw plant biomass. ^13^C-tracer analysis indicated when grown on raw corncob, the average rate of ^13^CO_2_ fixation for malic acid synthesis achieved 33.8 mg/L/h and the content of ^13^C atom in malic acid was up to 0.44 mol/mol malic acid (Fig. 6b), suggesting that 1 t of malic acid could be produced from 1.89 t of biomass with 0.14 t of CO_2_ fixation. Our results clearly show the synergy between lignocellulosic biomass conversion and CO_2_-fixation for producing organic acids.

As expected, the titer and yield of malic acid on corncob shown less than that on crystalline cellulose (Avicel), as more complicated structure and multiple components of plant biomass. Lignin acts as a barrier for depolymerization of plant cell wall and its degradation products are known to be toxic to microorganisms [46, 47]. Moreover, xylose occupies a large proportion of the components of plant biomass [9, 48]. However, the average rate of fixating and channeling CO_2_ into product malic acid in engineered strains on xylose was approximately 1.7-fold less than that on glucose (Fig. 4). Therefore, the further engineering of DMCC in *M. thermophila* is needed to improve overall bioconversion from plant biomass, such as speeding up pentose utilization and reducing the negative effect of lignin.

## Discussion

Plant biomass and CO_2_ have many desirable features as industrial raw material to decrease reliance on fossil fuels. Direct conversion of plant lignocellulose and fixing CO_2_ into the central carbon metabolism of industrial microbes are promising for cost-effective production of value-add compounds in biorefinery. The thermophilic and cellulolytic *M. thermophila* has been engineered to produce malic acid using raw plant biomass as the feedstock without addition of hydrolytic enzyme [10]. In this study, using *M. thermophila*, the DMCC system (direct microbial conversion of biomass with CO_2_ fixation), a novel strategy for biorefinery from plant cell wall and CO_2_, was constructed to convert plant biomass and CO_2_ into malic acid efficiently.

For fixing and channeling CO_2_ into carbon metabolism for producing value-added compounds, there are two different strategies. One approach is the integration of exogenous biosynthetic production pathways into naturally existing carbon-fixing organisms, such as cyanobacteria and algae. Autotrophic microbes have been engineered to produce chemicals and biofuels from CO_2_, such as 2,3-butanediol, lactic acid and malic acid [49–51]. However, production performance remains far below industrial feasibility. The other option is to equip heterotrophic fermentation strains with efficient CO_2_-fixation pathway. Recently, Calvin–Benson–Bassham (CBB) pathway was constructed in heterotrophic microbes to recycle released CO_2_ into central metabolic pathway for improved carbon efficiency [20, 22, 28] and even synthesize sugars and other major biomass components from CO_2_ [29]. An efficient energy supply is required for biological CO_2_ fixation. Engineered *E. coli* and *P. pastoris* could incorporate CO_2_ into cell components via heterologous CBB pathway, requiring reducing power and energy from the supplements, such as pyruvate, formate, and methanol [18, 29, 33]. Using sustainable plant biomass as energy source and actuate CO_2_-fixation would be a promising strategy for cost-effective bio-manufacturing. In addition, the main components of lignocellulose comprise glucose, xylose, and arabinose. Intermediate ribulose-5-phosphate of pentose catabolism via pentose phosphate pathway can serve as the substrate of PRK. In *E. coli* and *S. cerevisiae*, xylose and arabinose were used to drive CO_2_-fixation of the CBB cycle [17, 20, 32]. In this work, the CBB cycle enzymes were successfully introduced into the cellulohydrolytic fungus *M. thermophila* to producing CO_2_ fixation system, combined with native PYC module to produce malic acid using plant biomass and CO_2_ as the carbon sources. The proportion of carbon atoms from fixed CO_2_ in the total carbon of malic acid was significantly increased and the average rates of fixing and channeling ^13^CO_2_ into malic acid achieved 44.4 mg/L/h, 90.7 mg/L/h and 80.7 mg/L/h in strain CP-51, when grown on xylose, glucose and Avicel, respectively (Fig. 4). With raw corncob as the feedstock, the yield of malic acid produced by the final engineered strain Gal-1 achieved 0.53 g/g total plant biomass, representing a 10.4% increase over the highest reported yield (0.48 g/g) [9] and the content of ^13^C atom in malic acid was up to 0.44 mol/mol malic acid. Furthermore, this strategy of synergistic conversion of plant biomass and CO_2_ can be utilized for the production of other chemicals, such as fumaric and succinic acid.

Rapid utilization of all components of the hydrolysate of plant biomass is the prerequisite for efficient production of biochemical. However, due to the preference of microbes for glucose, pentose utilization is inhibited by glucose presented in the culture, which led to two-stage utilization of sugar mixture and low productivity of target products. It was suggested that d-glucose impairs the simultaneous utilization of pentose mainly by inhibition of pentose uptake [43]. Enhancement of pentose uptake can alleviate this inhibition and facilitate improved pentose utilization and elevated co-fermentation rates of hexose and pentose [44, 52]. Recently, transporter engineering and directed evolution have been used for rewiring substrate specificity to obtain glucose-insensitive xylose transporters [45, 53]. Herein, a glucose inhibition-free xylose transporter was integrated into engineered strain CP-51 for facilitating pentose utilization to actuate CO_2_-fixation of heterologous CBB pathway and improving co-fermentation rates of hexoses and pentoses for production of malic acid. Titer and yield of malic acid were increased to 76.1 g/L and 1.01 g/g carbon source, respectively, by conversion of a mixture of sugars derived from plant biomass. In addition, although xylose and arabinose are both pentose, there are dramatic differences in transcriptomic profiles in filamentous fungus when exposed to them in a previous study [9], indicating that that regulation network of xylose catabolism is different from that of arabinose catabolism. Here, it was also observed that titer of malic acid on xylose was obviously below that on arabinose. The exact molecular basis of the two pentose metabolism needs more investigation in the future.

In this study, CBB cycle enzymes, RuBisCO and PRK, were integrated into the metabolic network of the thermophilic fungus *M. thermophila* for enhanced fixation efficiency of CO_2._ A novel biorefinery system named DMCC was designed and constructed, which can produce 1 t of bulk chemicals (such as malic acid) using less than 2 t of plant biomass, accompanied by the fixation of 0.14 t CO_2_. This study provides a novel strategy for producing biochemicals and operating carbon neutral.

## Materials and methods

### Strains and culture conditions

*Myceliophthora thermophila* strain JG207 and its mutants were propagated on 1× Vogel’s minimal medium plates supplemented with 2% glucose at 35 °C to obtain conidia after 8 d, and corresponding antibiotic was added when needed for transformant screening.

*Escherichia coli* DH5α was employed for vector construction, and was cultivated in Luria–Bertani medium with 100 µg/mL ampicillin or 50 µg/mL kanamycin for plasmid selection.

### Plasmid construction

For the construction of plasmids overexpressing target genes, *cbbm* (GenBank no. X00286.1) from *Rhodospirillum rubrum* was codon-optimized on the basis of *Neurospora crassa* codon frequency (http://www.kazusa.or.jp/codon/), artificially synthesized, and inserted between the SpeI and BamHI sites of plasmid pAN52-PtrpC-neo-PMtgpdA [9] carrying the *neo* selectable marker to form overexpression plasmid PgpdA-cbbM-neo, using the NEB Gibson Assembly Kit. Similarly, codon-optimized *prk* (GenBank no. X07654.1) from *Spinacia oleracea*, under control of the strong constitutive promoter of *pdc* (Mycth_112121), was inserted between the BglII and BamHI sites of pAN52-PtrpC-neo-PMtgpdA to generate the vector Ppdc-prk-neo.

The strong constitutive promoter of *eif* (Mycth_2297659) was employed to efficiently overexpress pentose transporter genes. Site-directed mutation of sugar transporter gene *gal-2* from *S. cerevisiae* was performed using a fusion PCR strategy to generate Gal2M with mutation of residue N376 to phenylalanine (N376F). With the aid of the NEB Gibson Assembly Kit, the amplicons were ligated between the BglII and BamHI sites of pAN52-PgpdA-*bar* [10] to generate the corresponding plasmid P*eif*-gal2M-*bar*.

Plasmids for sgRNA expression were constructed as described previously [54]. Briefly, specific sgRNA target sites in target genes (*pck*, Mycth_2315623; *ldh*, Mycth_110317; and *pdc*, Mycth_112121) were identified using the sgRNACas9 tool [55] and the *M. thermophila* genome sequence and target gene sequences as the input. Oligos with no off-target probability were selected. The *M. thermophila* U6 promoter and a target-directed sgRNA fragment were amplified from the U6p-sgRNA plasmid [54], assembled by overlapping PCR, and cloned into blunt cloning vector pJET1.2 to generate the plasmids U6-pck-sgRNA, U6-ldh-sgRNA, and U6-pdc-sgRNA.

A vector carrying donor DNA was constructed to perform genomic modification. The 5′- and 3′-flanking fragments of *pck* were amplified from the *M. thermophila* genome. These fragments and selectable marker cassette P*trpC*-*neo* from plasmid p0380-neo [56] were assembled using the NEB Gibson Assembly Kit and cloned into pPK2BarGFPD digested with SpeI and EcoRV to generate the donor DNA sequence donor-pck-neo.

Codon-optimized *prk* and *cbbM* were amplified from plasmids Ppdc-prk-neo and PgpdA-cbbM-neo, respectively, using paired primers. *prk* and *cbbM* were knocked-in to the *ldh* and *pdc* loci of the *M. thermophila* genome, respectively, controlled by in situ promoters. 5′- and 3′-flanking fragments of the *ldh* and *prk* codon sequences were assembled to generate donor-ldh-prk. Similarly, 5′- and 3′-flanking fragments of *pdc* and *cbbM* codon sequences were assembled to generate donor-pdc-cbbM.

All vectors were constructed using *E. coli* DH5α and the target genes cloned into shuttle vectors were sequenced to verify the authenticity of the plasmid construction.

### *Myceliophthora* transformation

Polyethylene glycol-mediated transformation of *M. thermophila* protoplasts was performed as described previously [57]. For gene overexpression, 10 µg linearized plasmid were transformed into *M. thermophila* protoplasts. Putative transformants were selected on agar plates supplemented with corresponding antibiotics and confirmed via PCR amplification of the transgene with paired primers.

For multiple gene replacement involving the *pck*, *pdc*, and *ldh* loci, sgRNA and donor expression cassettes for *neo*, *prk*, and *cbbM* were mixed with Cas9-expression PCR cassette and co-transformed into strain JG207. Putative transformants were selected with 100 μg/mL G418 followed by sequential identification via PCR with paired primers.

### Malate-production medium

Shake-flask cultivation was performed with 50 mL of medium inoculated with mature spores at a final concentration of 2.5 × 10^5^ spores/mL in 250-mL Erlenmeyer flasks to evaluate the malic acid production capabilities of *M. thermophila*. The culture was incubated at 45 °C with shaking at 150 rpm and samples (1 mL) were taken at different intervals. Each liter of the cultivation medium contained 75 g of carbon source, 0.15 g of KH_2_PO_4_, 0.15 g of K_2_HPO_4_, 0.1 g of MgSO_4_·7H_2_O, 0.1 g of CaCl_2_·2H_2_O, 8 g of Bacto peptone, 1 mL of biotin (0.1 g/L), and 1 mL of trace element of Vogel’s salt, and was sterilized by autoclaving. Subsequently, sterilized CaCO_3_ was used as a neutralizing agent at a final concentration of 80 g/L to keep the pH at approximate 6.0. When corncob was used as the feedstock, mechanical pulverization was carried out as follows: corncob was chopped into pieces, pulverized by grinding mill, and then passed through the 80-mesh size sieve.

### Metabolite analysis

To detect organic acid titer in culture broth, 1 mL of 2 M sulfuric acid was added into 1-mL well-mixed sample in a 15-mL tube and the mixture was incubated at 80 °C for 30 min. The mixture was vortexed at intervals to resolve the malate adequately. Subsequently, 2 mL of distilled water was added, mixed, and an aliquot was used for metabolite analysis.

Malic acid titer was determined by high-performance liquid chromatography (HPLC) using an instrument (e2695; Waters, Manchester, United Kingdom) equipped with an Aminex HPX-87H column (Bio-Rad, Hercules, CA, USA) at 35 °C and a Waters 2489 UV detector at 40 °C; 5 mM H_2_SO_4_ was used as the mobile phase with a constant flow rate of 0.5 mL/min. Sugar concentrations were monitored with a Waters 2414 refractive index detector and an Aminex HPX-87P column (Bio-Rad) with distilled water as the mobile phase. Data analysis was performed using a Waters e2695 separation module.

### Enzyme assays

A 50-mL sample was poured into a Büchner funnel equipped with four pieces of gauze, washed with distilled water until most of the CaCO_3_ was removed, and subsequently collected. Then, mycelia were immediately homogenized in liquid nitrogen and ground into a powder in a prechilled mortar with a prechilled pestle. The paste was transferred into l mL phosphate-buffered saline (pH 7.4). After centrifugation for 10 min at 4 °C, clear supernatant was used for protein qualification and enzyme assay.

Protein concentration in supernatants was measured using a Bio-Rad protein assay kit. RuBisCO activity was measured by the modification of the method described by Xia et al. [17]. The reaction mixture containing 100 mM Tris (pH 7.4), 10 mM MgCl_2_, 20 mM NaHCO_3_, 10 mM KCl, 1 mM dithiothreitol, 2 mM oxaloacetate, 5 mM creatine phosphate, 10 U 3-phosphoglycerate kinase, 10 U glyceraldehyde 3-phosphate dehydrogenase, 10 U creatine phosphokinase, 0.2 mM NADH, and crude enzyme solution was incubated for 15 min at 30 °C. The assay was started by the addition of 0.5 mM ribulose-1,5-bisphosphate and was immediately monitored at 340 nm for 5 min. RuBisCO activity was defined as the amount of enzyme required to produce 1 nM product per min.

### Sugar uptake assays in *M. thermophila*

Strains were incubated in 100 mL of 1× Vogel’s medium containing 2% glucose at 45 °C for 18 h, and then washed three times in 1× Vogel’s salts without any carbon source. Subsequently, the mycelia were transferred to Vogel’s salts containing 0.5% sugar (glucose, xylose, or arabinose) for induction for an additional 4 h. After that, the mycelia were washed again as above and resuspended in uptake buffer [1× Vogel’s salts plus 10 mM sugar (glucose, xylose, or arabinose) and 10 μg/mL cycloheximide] for 20 min. The amount of residual sugar in the supernatant was determined and the fungal biomass was blotted dry and then completely dried at 105 °C to determine the dry weight for data normalization.

### Quantitative real-time PCR analysis

To assay copy numbers of genes ectopically inserted into the *M. thermophila* genome, fungal genomic DNA was extracted from transformants as described previously [58] and used as the template for real-time qPCR (RT-qPCR). Quantitative PCR was carried out with SYBR Green Realtime PCR Master Mix (Toyobo, Osaka, Japan) and a CFX96 real-time PCR detection system (Bio-Rad), according to the manufacturer’s instructions. The reaction mixture (with three replicates) included 1 μL of template DNA, 0.4 μL of each primer (10 μM), 10 μL of RNA-direct SYBR® Green Realtime PCR Master Mix, and 8.2 μL of H_2_O. The actin gene (MYCTH_2314852) was used as an internal control. The primers for each gene were optimized to obtain amplification efficiency between 95 and 105% and only one melting temperature on the melting curve. The primers used for RT-qPCR are listed in Additional file 1: Table S1.

### The assay of ^13^C-labeled malic acid

When ^13^C-tracer analysis was performed to confirm CO_2_ fixation during production of malic acid, Ca^13^CO_3_ was used as the neutralizer and provider of ^13^CO_2_. After the fermentation is over, samples were treated with sulfuric acid and diluted with the solution of acetonitrile–methanol–water (40:40:20, v:v:v) to a suitable concentration and centrifuged for 15 min at 12,000×*g*. An ABSciex 5600 TripleTOF liquid chromatography–tandem mass spectrometer (LC–MS/MS) equipped with a UHPLC LC-30A system was employed to determine the relative abundance of malate in supernatants and the chromatography used a zic-HILIC column (Merck, Germany). LC–MS/MS analysis was performed as described previously [9]. The ratio of the areas from the resulting multiple reaction monitoring peaks were used to assess the relative abundances of malic acid with ^13^C atom.

### Statistical significance tests

Unless otherwise noted, statistical significance was tested using a one-tailed homoscedastic (equal variance) *t*-test. All *p-*values were generated using Microsoft Excel 2013 (Microsoft Corporation). n.s. indicates no statistical significance; * represents a *p*-value < 0.05; ** represents a *p*-value < 0.01; and *** represents a *p*-value < 0.001.

## Supplementary Information

**Additional file 1: Table S1.** List of PCR primers used in this study.

**Additional file 2: Figure S1.** PCR analysis of the mutants of *M. thermophila* generated in this study. **Figure S2.** Copy number assay by RT-qPCR. *prk* and *cbbM* in the genome of strain CP-1; *prk* and *cbbM* in strain CP-51 genomic DNA; gal2M in the genome of strain Gal-1. The values and error bars represent means and standard deviations. **Figure S3.** Dry cell weight of strain CP-51 grown on xylose for 4 days. The values and error bars represent means and standard deviations of independent triplicate experiments, respectively. The values and error bars represent means and standard deviations. **Figure S4.** Titers of malic acid produced by strains JG207, CP-1, and CP-51, when grown on glucose and Avicel. Titer of malic acid was determined after 8 days of fermentation. The values and error bars represent means and standard deviations of independent triplicate experiments, respectively. The values and error bars represent means and standard deviations. **Figure S5.** Sugar utilization of strain Gal-1 when growth in Vogel’s minimal medium supplemented with single or multiple sugars derived from lignocellulose. **a** 40 g/L xylose; **b** 40 g/L arabinose; **c** 40 g/L glucose; **d** 40 g/L glucose and 20 g/L xylose; **e** 40 g/L glucose and 20 g/L arabinose; **f** 20 g/L xylose and 20 g/L arabinose; g 40 g/L glucose, 20 g/L xylose, and 20 g/L arabinose. The values and error bars represent means and standard deviations.

## Data Availability

All data generated or analyzed during this study are included in this published article and its Additional files.

## Abbreviations

CBP: Consolidated bioprocessing
CBB cycle: Calvin–Benson–Bassham cycle
PRK: Phosphoribulokinase
RuBisCO: Ribulose-1,5-bisphosphate carboxylase–oxygenase
rTCA pathway: Reductive tricarboxylic acid pathway
CRISPR: Clustered regularly interspaced short palindromic repeats
DMCC: Direct microbial conversion of biomass with CO_2_ fixation
PYC: Pyruvate carboxylase
