# Supplementary material for: Coordination of consolidated bioprocessing technology and carbon dioxide fixation to produce malic acid directly from plant biomass in Myceliophthora thermophila
Source: Biotechnol Biofuels. 2021 Sep 23;14:186. doi: 10.1186/s13068-021-02042-5 (PMC8461902; doi:10.1186/s13068-021-02042-5)
Supplement: Supplementary file 2 — Additional file 2: Figure S1. PCR analysis of the mutants of M. thermophila generated in this study. Figure S2. Copy number assay by RT-qPCR. prk and cbbM in the genome of strain CP-1; prk and cbbM in strain CP-51 genomic DNA; gal2M in the genome of strain Gal-1. The values and error bars represent means and standard deviations. Figure S3. Dry cell weight of strain CP-51 grown on xylose for 4 days. The values and error bars represent means and standard deviations of independent triplicate experiments, respectively. The values and error bars represent means and standard deviations. Figure S4. Titers of malic acid produced by strains JG207, CP-1, and CP-51, when grown on glucose and Avicel. Titer of malic acid was determined after 8 days of fermentation. The values and error bars represent means and standard deviations of independent triplicate experiments, respectively. The values and error bars represent means and standard deviations. Figure S5. Sugar utilization of strain Gal-1 when growth in Vogel’s minimal medium supplemented with single or multiple sugars derived from lignocellulose. a 40 g/L xylose; b 40 g/L arabinose; c 40 g/L glucose; d 40 g/L glucose and 20 g/L xylose; e 40 g/L glucose and 20 g/L arabinose; f 20 g/L xylose and 20 g/L arabinose; g 40 g/L glucose, 20 g/L xylose, and 20 g/L arabinose. The values and error bars represent means and standard deviations. [file 13068_2021_2042_MOESM2_ESM.pdf]

Additional file 2: Additional Figures

for

Coordination of consolidated bioprocessing technology and CO_2-fixation to produce malic acid directly from plant biomass in *Myceliophthora thermophila*

Li *et al.*

Strains	Primers	PCR analysis
CP-1	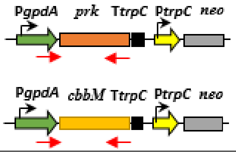	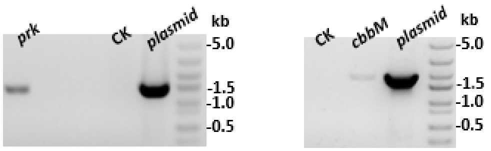
CP-51	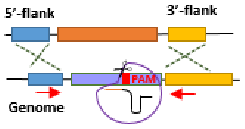	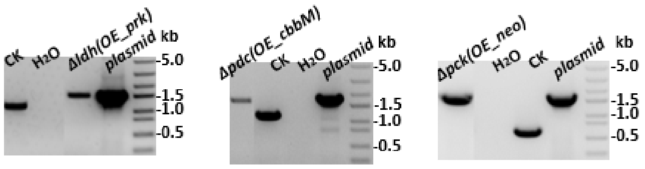
Gal-1	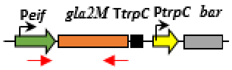	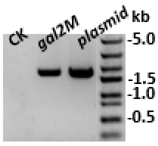
Gal-1Δ*pho*	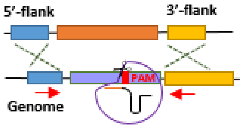	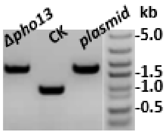

Figure S1. PCR analysis of the mutants of *M. thermophila* generated in this study.

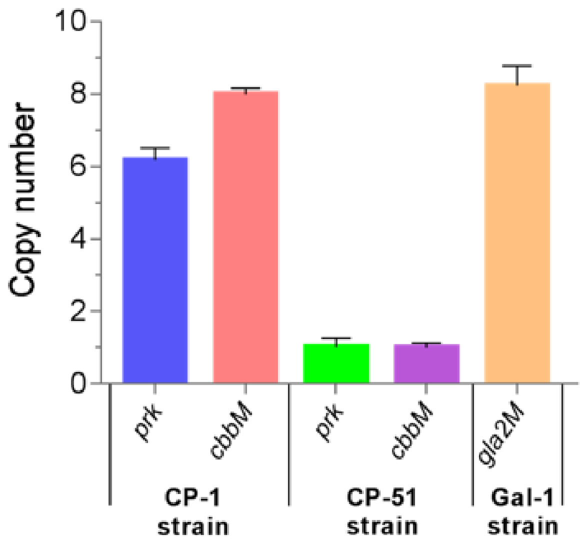

Figure S2. Copy number assay by RT-qPCR. *prk* and *cbbM* in the genome of strain CP-1; *prk* and *cbbM* in strain CP-51 genomic DNA; *gal2M* in the genome of strain Gal-1. The values and error bars represent means and standard deviations.

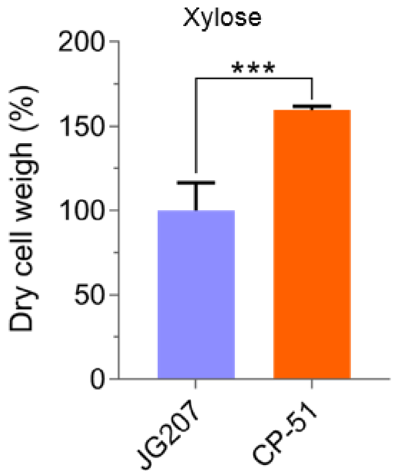

Figure S3. Dry cell weight of strain CP-51 grown on xylose for 4 days. The values and error bars represent means and standard deviations of independent triplicate experiments, respectively. The values and error bars represent means and standard deviations.

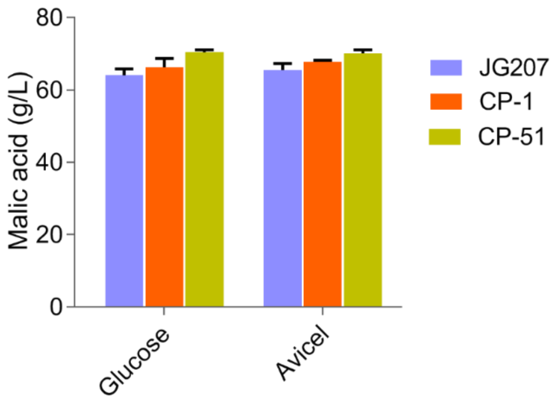

Figure S4. Titers of malic acid produced by strains JG207, CP-1, and CP-51, when grown on glucose and Avicel. Titer of malic acid was determined after 8 days of fermentation. The values and error bars represent means and standard deviations of independent triplicate experiments, respectively. The values and error bars represent means and standard deviations.

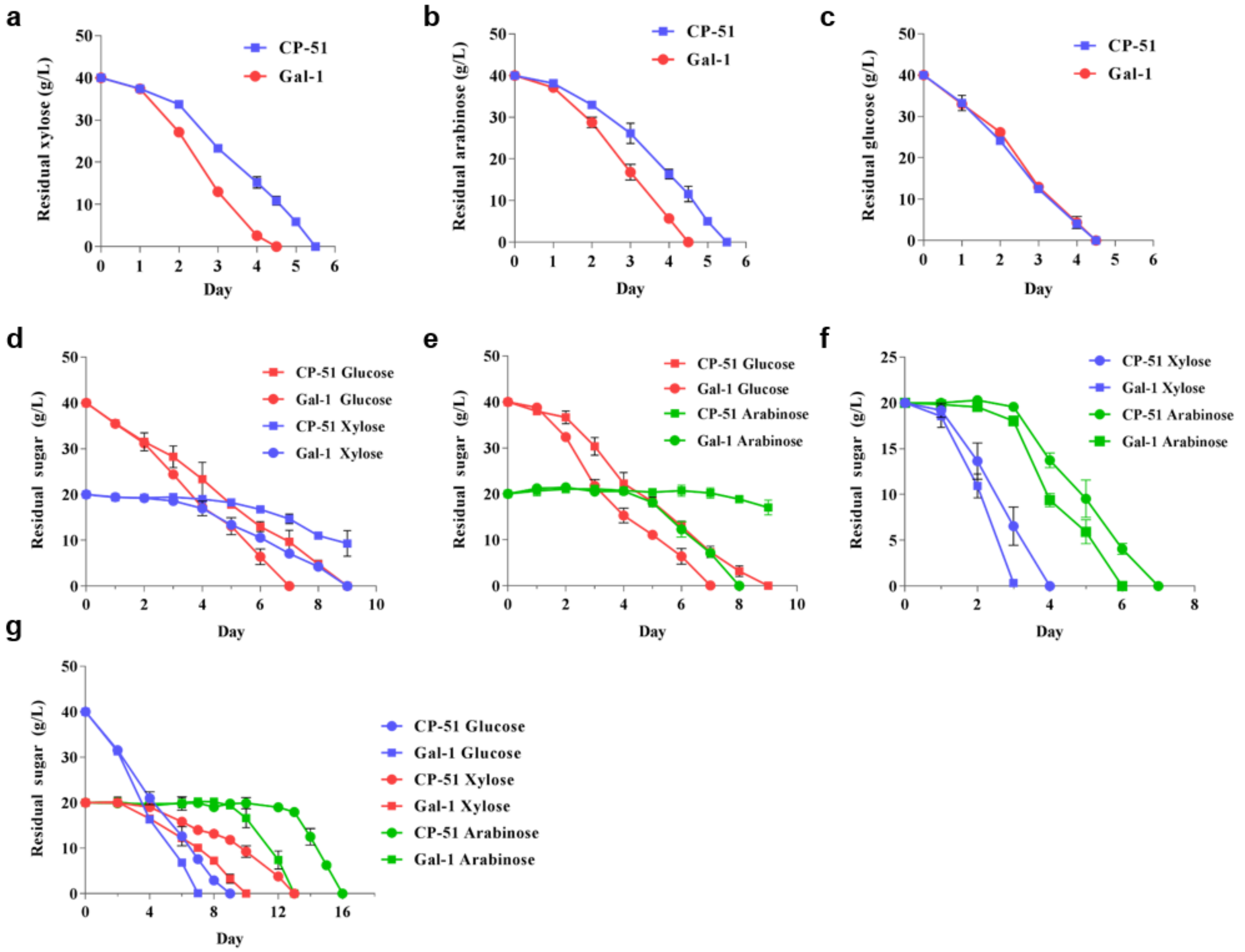

Figure S5 Sugar utilization of strain Gal-1 when growth in Vogel's minimal medium supplemented with single or multiple sugars derived from lignocellulose. **a** 40 g/L Xylose; **b** 40 g/L arabinose; **c** 40 g/L glucose; **d** 40 g/L glucose and 20 g/L xylose; **e** 40 g/L glucose and 20 g/L arabinose; **f** 20 g/L xylose and 20 g/L arabinose; **g** 40 g/L glucose, 20 g/L xylose, and 20 g/L arabinose. The values and error bars represent means and standard deviations.
